# Differentiating Between Youth with a History of Suicidal Thoughts, Plans, and Attempts

**DOI:** 10.1007/s12310-023-09575-0

**Published:** 2023-02-24

**Authors:** Alannah Shelby Rivers, Payne Winston-Lindeboom, Tita Atte, Perri Rosen, Matt Wintersteen, Nicole Kathleen Watkins, Allen Tien, Guy Diamond

**Affiliations:** 1 Center for Family Intervention Science, College of Nursing and Health Professions, Drexel University, Philadelphia, PA USA; 2 Pennsylvania Garrett Lee Smith Youth Suicide Prevention Grant, Harrisburg, PA USA; 3 Department of Psychiatry and Human Behavior, Sidney Kimmel Medical College, Thomas Jefferson University, Philadelphia, PA USA; 4 Medical Decision Logic, Inc, Baltimore, MD USA; 5 Social Work, Psychology, and Philosophy, Texas Woman’s University, Denton, TX USA

**Keywords:** Adolescent, Depression, Mental health, Students, Suicidal thoughts

## Abstract

Limited research has examined factors distinguishing between patterns of adolescent suicidal thoughts and behaviors. The current study examined demographic, school, family, and mental health differences across patterns identified by Romanelli and colleagues (2022): history of thoughts only, plans with thoughts, attempt with thoughts and/or plans, and attempt without thoughts. The current study includes 4,233 students (*M*_*age*_ = 14.65 years, *SD* = 2.06) with a history of suicide risk referred to school Student Assistance Program teams. The sample was approximately 60.7% female, 59.8% White (16.0% Black, 15.4% multiracial, 8.8% other), and 14.4% Hispanic. Results indicated that the “attempt without thoughts” group was small with no differentiating characteristics. However, membership in the other three groups was predicted by demographic, school, family, and mental health factors. These results support the importance of examining suicidal thoughts, plans, and attempts as distinct indicators and assessing key biopsychosocial factors. Further research could improve how behavioral health systems identify at risk youth.

## Introduction

More than 700,000 people worldwide die from suicide each year (World Health Organization, 2021), and suicide continues to rise in the United States (Centers for Disease Control and Prevention, 2021a, 2021b). It is the 10th leading cause of death overall (World Health Organization, 2015) and the 2nd leading cause of death among youth 12 to 24 years old in the United States (Heron, 2018; Stone et al., 2018; World Health Organization, 2015). Adolescent suicide is at its highest rate since 2000 (Centers for Disease Control and Prevention, 2021a, 2021b). In 2019, one in 11 high school students made an attempt, 1 in 6 made a plan, and 1 in 5 had suicidal thoughts (Ivey-Stephenson, 2020). Moreover, during the COVID-19 pandemic, emergency department visits for suicide attempts increased in middle and high school aged youth (Ridout et al., 2021; Yard et al., 2021).

### Suicide Prevention in Schools

School decision-makers have a vested interest in the well-being of the students, and nowhere is this more apparent than in the case of suicide. In general, suicides have a devastating and far-reaching impact on the surrounding community. One study of community impact of suicide deaths estimated that each suicide has the potential to affect up to 135 individuals, with as many as 25 reporting severe long-term effects (Cerel et al., 2016). For adolescents in schools, the impact may be even more widespread, as *suicide contagion*, the spread of suicidal thoughts and behavior, is more pronounced in this age group (Lake & Gould, 2014). For example, adolescents who lose a close friend through suicide are more than three times as likely to report suicidal thoughts (Song et al., 2015). Therefore, suicide prevention and early intervention are essential for ensuring the well-being of not only the targeted students but also the entire school community.

Because of this issue, and because schools are widely viewed as a natural setting for identifying youth in need of mental health services (Adelman & Taylor, 2007; Weist & Murray, 2008), mental health programs and suicide prevention efforts have become increasingly common in schools. Many of these suicide prevention efforts have focused on screening students for mental health and suicide risk, either in a targeted or universal fashion (Substance Abuse and Mental Health Services Administration, 2019; Singer et al., 2019). Research has been encouraging on the potential effectiveness of this approach, with studies suggesting that youth are generally willing to self-disclose mental health problems, including suicidal thoughts and behaviors, when directly asked (Hilt et al., 2018; Mann et al., 2005).

### Distinct Factors Related to Suicidal Thoughts and Attempts

However, screening is only the first step to suicide prevention. Unfortunately, when screening occurs, it is not always clear when further intervention is needed. To help reduce suicide death, more research is needed on suicide risk that may help inform clinical decisions. For example, it is important to understand what levels of suicidal thoughts and behaviors (e.g., thoughts, plans, attempts) may represent meaningfully distinct groups of students. It is also important to understand other factors that could be assessed in a broad psychosocial screener and that may be linked to suicidal thoughts and behaviors. Although there is a wealth of research identifying factors distinguishing suicidal from non-suicidal youth, it may be even more crucial to understand psychosocial factors that lead an individual to go from suicidal thoughts to actions. Only one-third of those who report suicidal thoughts end up developing a plan, and even fewer make an attempt (Borges et al., 2010; Mars et al., 2019). Identifying risk factors associated with these different risk levels might both improve individual assessment of risk and clinical decision-making, and lead to improved knowledge on interventions to prevent the escalation of suicidal thoughts into behaviors (Glenn & Nock, 2014; Klonsky & May, 2014).

Research on factors that distinguish between those with suicidal thoughts only and those who attempt suicide but do not die has found these groups are distinct in several ways. For example, family cohesion has emerged as a protective factor, keeping adolescents with thoughts from later engaging in a suicide attempt (Sun et al., 2020). Similarly, bullying and academic struggles have been found to differentiate those with thoughts alone from those who planned or attempted suicide (Adewuya & Oladipo, 2020; Romanelli et al., 2022). Other home and school factors (e.g., family conflict, low parental monitoring, having close friends at school) have been associated with both thoughts and attempts (Adewuya & Oladipo, 2020; DeVille et al., 2020; Govender et al., 2013; Marraccini & Brier, 2017; Tang et al., 2009), but have been infrequently investigated as a differentiating factor. Finally, these two groups differ in terms of mental health risk factors. Those who attempt report far more psychopathology (e.g., depression, traumatic distress, substance use), and both groups report more symptoms compared to non-suicidal individuals (Mars et al., 2019; May & Klonsky, 2016).

### Additional Patterns of Suicidal Thoughts and Behavior

However, little research has examined suicide patterns beyond the thoughts-attempt distinction (Borges et al., 2010). For example, most suicide screeners ask about patients who have a suicide plan, but few studies examine if that group is meaningfully distinct from those with thoughts or those who attempt. Moreover, some individuals may make an attempt without thoughts or plans; unplanned suicide attempts make up about 30% of all attempts (Borges et al., 2010). These are important distinctions that are not always addressed when comparing suicidal individuals (May & Klonsky, 2016).

One recent study extended this research by examining four unique patterns of suicidal thoughts and behaviors over the past year (Romanelli et al., 2022). Using a large, national sample of students in schools, Romanelli et al. (2022) found significant distinctions between these patterns based on demographic characteristics (age, sex, and race/ethnicity), bullying, depressive symptoms, history of sexual violence, and substance use. Use of e-cigarettes and illicit substances and misuse of prescription drugs differentiated those with suicidal plans and thoughts from thoughts alone. Furthermore, students with “unplanned” attempts (without reported thoughts or plans) were less likely to report common correlates of suicidal behavior, such as bullying and depressive symptoms, and were more likely to be Black and male compared to those with attempts accompanied by thoughts and/or plans. The authors highlighted the need for suicide assessments to evaluate additional factors that may help differentiate adolescents with suicidal thoughts, plans, and behaviors. Given the unique nature of the Romanelli et al. (2022) study and the need to replicate their findings, as well as the existing widespread assessment of these three indicators (thoughts, plans, attempts) in schools, the current study examined these same patterns. Therefore, in the current, exploratory study, a sample of middle and high school students who were referred for further mental health evaluation were used to explore differences in demographic and psychosocial characteristics between four suicide history patterns: thoughts-only, thoughts and plans, attempt with thoughts and/or plans, and attempt without thoughts or plans. Following a description of group differences, factors uniquely distinguishing each pattern were examined.

## Materials and Methods

### Participants and Procedure

The sample derives from a dataset of 12,760 middle and high school students referred to school Student Assistance Program (SAP) teams (Substance Abuse and Mental Health Services Administration, 2019). Of these, 4233 students reported having suicidal thoughts and/or behaviors at some time in their life. All subsequent analyses were based on this sample of 4,233 students (*M*_*age*_ = 14.65 years, *SD* = 2.06). The sample was 60.7% female, 37.1% male, and 1.3% other genders, with 1.5% identified as transgender. In terms of race, 59.8% identified as White, 16.0% as Black, 15.4% as mixed race/multiracial, 6.5% as other races, and 2.3% as Asian. Approximately 14.4% identified as Hispanic.

The Student Assistant Program (SAP) is required in every school in Pennsylvania to address barriers to learning. When a youth is identified (by school personnel or another individual) as having barriers to academic success and well-being, they are referred to a school-based team, who may refer the student to a SAP liaison from a community behavioral health agency if there are concerns about an underlying issue for which the student may need a screening or assessment. These assessments are often completed at the school and are used to identify school and/or community-based recommendations to help students overcome recognized barriers. Parents and/or legal guardians provide consent for student participation in the SAP process and for any screening or assessment by a SAP liaison; the current data come from SAP liaison agencies where guardians provided permission for the de-identified data to be shared with Drexel University for program evaluation and research purposes. The sample for this study is not a universal sample of school students, but a referred sample of at-risk youth who were universally screened for suicide risk by SAP agencies utilizing the BHS. All procedures for this study were approved by the Drexel University Institutional Review Board.

### Measures

#### Behavioral Health Screen

The Behavioral Health Screen (BHS; Diamond et al., 2010) is an adolescent self-report tool that includes questions covering 15 domains: demographics, medical, school, family, safety, substance use, sexual risk, nutrition and eating, anxiety, depression, suicide and self-harm, psychosis, trauma, bullying, and gun access. There are 61 main questions and 46 follow-up questions with skip outs if critical items are not endorsed. The tool takes between 9 and 14 min to administer. The BHS has strong psychometric characteristics (Bevans et al., 2012; Diamond et al., 2010, 2017), and is embedded in bhworks, a multicomponent software platform that assists with consenting, screening, assessment, safety planning, referral, and program evaluation. Bhworks is hosted by mdlogix, a health informatics software engineering company (bh-works.com). Because it is a web-based tool, the platform instantly scores the data and generates a report to help guide the clinical interview. The BHS has been used in schools, emergency departments (Herres et al., 2018), primary care (Diamond et al., 2017), and a residential facility (Ruan-Iu et al., 2022a, 2022b).

##### Lifetime Suicide Risk

Three dichotomous BHS-Suicide items were used to categorize students by lifetime suicide risk: suicidal thoughts, plans, and attempts.

##### Symptom Scales

The BHS symptom scales were used to assess mental health symptoms: depression, anxiety, traumatic distress, eating disorder, and substance use. All scales are brief, between four to five items. Most items are answered on a three-point Likert scale (e.g., Never, Sometimes, Often), although some (including all items for traumatic distress and substance use) are dichotomous. Cronbach’s alphas were 0.79 for depression, 0.76 for anxiety, 0.76 for traumatic distress, 0.62 for eating disorder, and 0.64 for substance use in the current sample. Symptom scales were summed and then dichotomized based on previously established and validated risk scores (Bevans et al., 2012), except for depression which was summed and then divided into four risk categories (no symptoms, mild symptoms, moderate symptoms, severe symptoms) based on previously established cutoffs.

##### School and Home Factors

Other risk factors were drawn from the school (skipping class, grades dropping, physical, verbal, and cyberbullying), safety (home violence, neighborhood violence, gun access, assault by a family member), and family (parental criticism, arguing in home, concern about family member substance use, going to family members for support, and parental monitoring) domains. All of these indicators were single items. Most of these items are answered on a three-point Likert scale (e.g., Never, Sometimes, Often), although some are dichotomous and one item (family criticism) is answered on a five-point Likert scale. For the current analysis, items were dichotomized (e.g., 0 = never, 1 = sometimes or often).

### Approach to Analysis

Initial groups were based on Romanelli and colleagues’ (2022) four-group classification: 1) those with a history of thoughts only, 2) plans with thoughts, 3) attempt with thoughts and/or plans, and 4) attempt without thoughts or plans. However the last group (attempt without thoughts or plans) was extremely small (*n* = 58), and initial analyses did not find evidence of differentiation from other groups. Therefore, this group was excluded from the analyses. An additional group not examined by Romanelli and colleagues (2022), those with a history of plans without thoughts, was also examined; this group size was similarly small (*n* = 48), and subsequently excluded. Therefore, the current study focuses on three lifetime risk groups: thoughts-only (*n* = 2054), thoughts and plans (*n* = 585), and attempt with thoughts and/or plans (*n* = 1437).

Chi-square tests were used to examine omnibus differences across the three groups (except for differences in continuous age, tested with the F statistic). When the omnibus test was significant, z-tests with Bonferroni corrections were used to examine differences between the individual groups. Then, all variables were entered as predictors in a multinomial logistic regression predicting group assignment. 0.4% of students had missing data on one or more variables; listwise deletion was used to handle this small proportion of missing data.

## Results

Proportions of variables across groups are shown in Table 1. Regarding demographics, there were significant differences across the three groups in race, gender, ethnicity, and age, but not in prevalence of transgender youth. Post hoc analyses indicated there were no significant demographic differences between the “thoughts” and “thoughts and plans” groups. On the other hand, the “attempt” group had significantly higher proportions of female, Hispanic, Black, and multiracial students than the other two groups. The “attempt” group was also slightly older than “thoughts-only” (although all groups had a mean age between 14 and 15 years) and had a lower proportion of Asian students than the “thoughts and plans” group. The prevalence of other-race identities did not differ across groups.

**Table 1:** Proportions across suicide risk groups

Variable	Group			Omnibus test
	1	2	3	
*Demographics*				
Gender				57.87***
Female	56.2^a^	59.3^a^	68.3^b^	
Male	41.7^a^	37.9^a^	29.2^b^	
Other	2.0^a^	2.7^a^	2.5^a^	
Transgender	1.4	1.7	1.7	0.69
Race				34.98***
White	62.3^a^	62.9^a^	55.5^b^	
Asian	2.6^a,b^	3.4^b^	1.5^a^	
Black	14.9^a^	12.3^a^	18.2^b^	
Mixed Race	14.0^a^	15.6^a^	17.7^b^	
Other	6.2^a^	5.8^a^	7.1^a^	
Hispanic	13.2^a^	13.5^a^	16.3^b^	6.81*
Age (mean)	14.58^a^	14.61^a,b^	14.76^b^	3.26*
*School*				
Skips class often	7.2^a^	9.4^a,b^	11.8^b^	21.22***
Grades declining	39.1^a^	46.2^b^	38.2^a^	11.75**
Verbal bullying	19.3^a^	22.4^a,b^	24.3^b^	12.89**
Physical bullying	2.5^a^	2.2^a,b^	4.2^b^	10.08**
Cyberbullying	2.1^a^	2.7^a,b^	4.9^b^	22.66***
No friends at school	8.7	10.3	10.6	4.17
*Home*				
Frequent violence at home	5.3^a^	8.2^b^	7.3^b^	9.62**
Neighborhood violence	7.8^a^	9.9^a,b^	12.0^b^	9.09*
Access to gun	7.3^a^	8.9^a,b^	11.3^b^	16.84***
Parents highly critical	34.4	38.5	35.4	3.26
Frequent arguing in home	29.1^a^	36.8^b^	33.1^b^	14.70***
No adult support	36.5^a^	44.3^b^	41.5^b^	15.75***
Parents never know location	2.4^a^	1.9^a^	4.3^b^	13.23**
*Mental Health*				
Anxiety	82.1^a^	89.1^b^	87.2^b^	26.49***
Depression				70.48***
None	5.1^a^	1.4^b^	2.8^b^	
Mild	18.5^a^	12.8^b^	12.5^b^	
Moderate	11.3^a^	11.5^a,b^	8.4^b^	
Severe	65.1^a^	74.4^b^	76.4^b^	
Traumatic distress	66.7^a^	74.5^b^	77.4^b^	50.36***
Eating disorder	9.0^a^	13.3^b^	16.6^b^	46.87***
Substance use	6.4^a^	6.5^a^	11.8^b^	35.93***

Group 1 = thoughts-only, 2 = thoughts and plans, 3 = attempt with thoughts and/or plans. Omnibus tests were conducted using χ^2^, except for continuous age tested with *F*. Groups with the same superscript did not significantly differ at *p* < 0.05 (after Bonferroni correction).

Regarding school variables, there were significant differences across the three groups in all three types of bullying, grades declining, and skipping class, but not in the presence of friends at school. Compared to “thoughts-only”, those with a history of attempt were significantly more likely to report skipping class often and experiencing bullying (physical, verbal, and cyberbullying). The “thoughts and plans” group did not significantly differ from the other two in any of these variables, with proportions generally falling in between those of the other two groups. For example, whereas 7.2% of the “thoughts-only” group reported frequently skipping class, 9.4% of the “thoughts and plans” group did so, compared to 11.8% of the “attempt” group. However, the “thoughts and plans” group was significantly more likely to report declining grades than either of the other groups; nearly half of this group (46.2%) reported a significant decline in their grades.

Regarding home variables, there were significant differences across the three groups in violence at home and in the neighborhood, gun access, arguing in the home, adult support, and having parents know the student’s location (parental monitoring). Two other home variables were considered: concern about substance use in the family and experiencing physical assault from a family member. However, no youth in the sample reported either, so these variables could not be included. There were no differences across groups in perceived parental criticism. Compared to the “thoughts-only” group, the other two groups had higher reported prevalence rates of home violence, arguing in the home, and a lack of adult support. The “attempt” group was also more likely to report neighborhood violence and gun access, compared to the “thoughts-only” group; 11.3% of those reporting a past attempt had access to a gun. Once again, the “thoughts and plans” group did not significantly differ from either group on these two variables, with intermediate rates of endorsement (e.g., 9.9% reporting frequent neighborhood violence compared to 7.8% for thoughts-only and 12.0% for attempt). The “attempt” group was also unique in reporting low parental monitoring compared to the other two groups, although this was still relatively rare (less than 5% of the “attempt” group said their parents “never” knew their location).

Regarding mental health variables, there were significant differences between the three groups in scoring into risk ranges for anxiety, depression, traumatic distress, substance use, and eating disorder. Both the “thoughts and plans” and “attempt” groups were more likely to report significant symptoms of anxiety, traumatic distress, and eating disorder compared to “thoughts-only”. For example, two-thirds (66.7%) of the “thoughts-only” group had significant symptoms of traumatic distress, compared to approximately three-quarters of the other two groups (74.5% and 77.4% of thoughts and plans and attempt groups, respectively). Depression symptoms, which were stratified into risk categories, indicated that those in the “thoughts-only” group were significantly more likely to report either no significant symptoms or mild symptoms compared to the other two groups, and were more likely to report moderate symptoms compared to the “attempt” group. On the other hand, severe depressive symptoms were substantially more common in the “thoughts and plans” and “attempt” group, with approximately three-quarters of each of these groups (74.4% and 76.4%, respectively) endorsing severe symptoms. The “attempt” group was also more likely to have substance use symptoms compared to the other groups (with 11.8% reporting such symptoms, compared to 6.4% and 6.5% in the other two groups).

### Multinomial Logistic Regression

Results from the Nagelkerke’s pseudo-R^2^ suggested the full set of predictors explained approximately 8.3% of the variance between groups. Odds ratios are shown in Table 2.

**Table 2:** Odds ratios

Variable	Thoughts + Plans (vs. Thoughts)	Attempts (vs. Thoughts)	Attempts (vs. Thoughts + Plans)
*Demographics*			
Gender			
Female	1.03 [0.84, 1.28]	1.63*** [1.39, 1.92]	1.59*** [1.28, 1.97]
Other	1.08 [0.40, 2.76]	1.78 [0.86, 3.69]	1.48 [0.62, 3.51]
Transgender	1.12 [0.35, 3.63]	1.16 [0.48, 2.72]	0.95 [0.33, 2.73]
Race			
Asian	1.28 [0.73, 2.17]	0.61 [0.36, 1.05]	0.53 [0.28, 1.00]
Black	0.76 [0.56, 1.03]	1.32** [1.07, 1.62]	1.70*** [1.26, 2.29]
Mixed Race	0.97 [0.70, 1.34]	1.10 [0.86, 1.40]	1.21 [0.89, 1.64]
Other	0.85 [0.51, 1.45]	1.22 [0.86, 1.76]	1.39 [0.88, 1.64]
Hispanic	0.96 [0.70, 1.33]	1.06 [0.84, 1.34]	1.10 [0.79, 1.53]
Age	1.00 [0.95, 1.05]	1.05* [1.01, 1.09]	1.05 [0.99, 1.10]
*School*			
Skips class often	1.24 [0.88, 1.76]	1.37* [1.06, 1.77]	1.14 [0.82, 1.60]
Grades declining	1.17 [0.96, 1.44]	0.87 [0.75, 1.02]	0.73** [0.59, 0.89]
Verbal bullying	1.01 [0.78, 1.31]	1.22 [0.93, 1.37]	1.09 [0.85, 1.40]
Physical bullying	0.87 [0.42, 1.73]	1.31 [0.82, 2.10]	1.76 [0.92, 3.35]
Cyberbullying	1.16 [0.59, 2.31]	1.82* [1.14, 2.93]	1.54 [0.85, 2.78]
No friends at school	1.07 [0.78, 1.51]	1.11 [0.86, 1.42]	1.04 [0.75, 1.44]
Home			
Frequent violence at home	1.29 [0.87, 1.90]	1.00 [0.73, 1.38]	0.83 [0.57, 1.20]
Neighborhood violence	1.13 [0.93, 1.41]	1.17 [1.00, 1.37]	0.97 [0.79, 1.19]
Access to gun	1.30 [0.92, 1.85]	1.46** [1.13, 1.90]	1.21 [0.86, 1.69]
Parents highly critical	1.02 [0.83, 1.26]	0.88 [0.75, 1.03]	0.84 [0.68, 1.04]
Frequent arguing in home	1.17 [0.95, 1.46]	1.02 [0.87, 1.21]	0.86 [0.70, 1.08]
No adult support	1.28* [1.04, 1.57]	1.14 [0.98, 1.33]	0.88 [0.72, 1.08]
Parents never know location	0.75 [0.38, 1.50]	1.41 [0.91, 2.18]	2.10* [1.07, 4.10]
Mental Health			
Anxiety	1.18 [0.83, 1.65]	0.90 [0.70, 1.14]	0.78 [0.55, 1.11]
Depression	1.16* [1.01, 1.34]	1.14* [1.02, 1.26]	0.96 [0.83, 1.11]
Traumatic distress	1.23 [0.97, 1.55]	1.39*** [1.16, 1.66]	1.18 [0.92, 1.50]
Eating disorder	1.16 [0.85, 1.60]	1.46** [1.16, 1.84]	1.20 [0.90, 1.61]
Substance use	0.91 [0.61, 1.35]	1.79*** [1.38, 2.34]	2.00*** [1.36, 2.94]

Group 1 = thoughts-only, 2 = thoughts and plans, 3 = attempt with thoughts and/or plans. 95% confidence intervals are in brackets. Reference groups were male, White, non- Hispanic/unsure, cisgender.

*p < 0.05, **p < 0.01, ***p < 0.001.

Two factors uniquely predicted membership in the “thoughts and plans” group compared to “thoughts-only”: parental support and depression. Specifically, students who “never” turned to adult family members for support had 28% higher odds to be in the “thoughts and plans” group after accounting for other factors. Moreover, with each successive risk level in depressive symptoms, students had 16% higher odds to be in the “thoughts and plans” group.

Ten unique factors predicted membership in the “attempt” group compared to “thoughts-only”: gender, race, age, skipping class, cyberbullying, gun access, depression, traumatic distress, eating disorder, and substance use. Girls had 63% higher odds to be in the “attempt” group compared to boys, and Black students had 32% higher odds compared to White students. For each year of age, there was a 5% increase in odds of being in the “attempt” group. Students who “often” skipped class had 37% higher odds to be in the attempt group compared to thoughts-only, and students who were frequently cyberbullied had 82% higher odds. The most striking results were in mental health symptoms. In addition to depression, which increased odds of being in the attempt group by 14% per risk category, all other symptoms besides anxiety uniquely increased odds of attempt group membership: traumatic distress (39%), eating disorder (46%), and substance use (79%).

Finally, five factors uniquely predicted membership in the “attempt” group compared to “thoughts and plans”: gender, race, lack of declining grades, lack of parental monitoring, and substance use. Girls had 59% higher odds of being in the “attempt” group compared to boys, and Black students had 70% higher odds compared to White students. Those with declining grades were more likely to be in the “thoughts and plans” group than in the “attempt” group (27% higher odds). The odds of being in the “attempt” group were doubled for those with significant substance use symptoms and were 2.1 times higher for those whose parents “never” knew their location.

## Discussion

The current study utilized a large sample of students referred to school SAP teams, to investigate factors differentiating youth with three patterns of lifetime suicidal thoughts and behavior: thoughts only, thoughts and plans, and attempts (with plans and/or thoughts). There were substantial differences between groups in demographic, school, family, and mental health variables. These findings have important implications for understanding factors related to suicidal thoughts and behaviors in youth and may inform triage decisions and intervention.

### Implications

These results support the utility of examining suicidal thoughts, plans, and attempts as distinct indicators of suicide risk. In line with previous research, there were substantial differences between those with histories of thoughts and attempts (Adewuya & Oladipo, 2020; Borges et al., 2010; May & Klonsky, 2016), emphasizing the importance of examining thoughts and behaviors separately. This study also adds to the small but growing body of literature finding that those with suicidal plans in combination with thoughts may be unique from those with thoughts alone (Romanelli et al., 2022). Although there were no demographic differences between the “thoughts-only” and “thoughts and plans” groups, youth with a history of suicidal plans were more likely to report declining grades, violence and arguing at home, and lack of family support, alongside symptoms of anxiety, severe depression, traumatic distress, and eating disorders. After accounting for all factors, those with thoughts and plans were uniquely distinguished by two factors: lack of family support and increased depressive symptoms. These findings have implications for clinical decision-making, suggesting that youth reporting suicidal plans may warrant a more in-depth assessment before triage compared to those with thoughts alone.

There were some notable demographic factors distinguishing students with a history of attempt from the other two groups. Black and/or female students were particularly likely to have attempted suicide (Hispanic and multiracial students were also at increased risk, but this difference was not significant after accounting for other factors). This is consistent with findings that, despite decreases in adolescent suicidal thoughts over the past 30 years, Black adolescent suicide attempts have actually *increased* over time (Lindsey et al., 2019). This troubling pattern has been tentatively attributed to multiple factors, including exposure to racism on both individual and societal levels (e.g., exposure to coverage of police violence against unarmed Black men), although more research is needed (Sheftall & Miller, 2021). Moreover, previous research has consistently found that female individuals (both youth and adults) are at heightened risk of suicidal thoughts and behaviors (Auerbach et al., 2015; Borges et al., 2010; Mars et al., 2019; May & Klonsky, 2016), although male individuals are more likely to die by suicide (due largely to differences in method of attempt; World Health Organization, 2021). This disparity may be attributed to the nearly doubled risk of depression in girls and women across the lifespan, which itself appears multifactorial (biological and sociocultural; Hyde & Mezulis, 2020). Therefore, programs targeting Black and female students may be especially important to prevent possible escalation of suicidal thoughts and plans into attempts. Further research on Black youth suicide risk in particular is needed, as outlined, in line with the priorities described in a recent paper by Sheftall and Miller (2021) and in the increased grant funding opportunities out of NIMH encouraging more suicide research with this population.

Second, school and home risk factors also distinguished the three groups. Of the 13 risk factors examined, 11 were associated with group membership (only school friendships and parental criticism failed to distinguish between groups). This is particularly notable because, although these types of factors have often been explored in research seeking to distinguish youth with suicide risk from non-suicidal youth (e.g.,Adewuya & Oladipo, 2020; DeVille et al., 2020; Marraccini & Brier, 2017), there is still need for research examining whether these factors are associated with more severe suicidal thoughts and behaviors. In particular, a lack of family support uniquely differentiated those reporting suicidal plans from those with thoughts alone. Skipping class, experiencing cyberbullying, and having access to a gun differentiated those reporting a history of suicidal attempts from those with thoughts alone. Finally, a lack of parental monitoring uniquely distinguished those with a history of suicidal attempts from those with thoughts and plans (these students were also less likely to report declining grades). In addition to potentially informing triage decisions, these associated factors suggest possible avenues for intervention to avoid escalation of suicidal thoughts. For example, at the school level, early identification programs targeting students frequently missing class could be beneficial. Moreover, given the importance of multiple family variables, interventions that focus on the family unit may be especially relevant for reducing risk of escalation (e.g., Attachment-Based Family Therapy; Diamond et al., 2016).

Finally, a wide spectrum of mental health symptoms was associated with increasing levels of suicide risk. Consistent with previous research (Mars et al., 2019), students with a history of plans and/or attempts were consistently distinguished by higher levels of anxiety, depression, traumatic distress, eating disorder, and (for those with a history of attempts) substance use. Depressive symptoms uniquely distinguished those with thoughts and plans from those with thoughts alone. Even more notably, all symptoms except anxiety were *uniquely* associated with a higher likelihood of attempt history compared to thoughts alone, and those who reported substance use had double the odds of having a past attempt compared to thoughts and plans. These findings further underscore the importance of comprehensive mental health assessment, beyond depressive symptoms alone, to aid in clinical decision-making and risk assessment for those reporting suicidal thoughts and behaviors.

### Limitations and Future Research

There are several limitations to the current study. First, the data are cross-sectional. Longitudinal research is needed to determine whether these factors predict future suicide risk, rather than differentiating youth at the time of their survey responses. Second, these data come from a sample of students referred to a school-based team (the majority were initially referred for behavioral concerns before completing a more thorough assessment), who may differ from non-referred students in several important ways (e.g., presence of externalizing symptoms). Replicating these results with other populations (including children who were not initially referred) would provide more generalizability. Third, these findings reflect only lifetime suicidal thoughts and behaviors. Current suicidal thoughts and behaviors are also measured by the BHS, but the subsample of adolescents endorsing current suicide risk was insufficiently sized for the current investigation. In this dataset, only 7% of youth reported current suicidal thoughts or behaviors, but 25% endorsed lifetime thoughts or behaviors (including those with current thoughts or behaviors). Endorsement of lifetime suicidal thoughts, plans, or behaviors identified youth struggling with risk factors that may result in an increased risk of a suicide attempt. Future research with larger samples should examine factors related specifically to current thoughts and behaviors. Finally, not all suicide patterns could be explored due to small cell sizes. This is particularly important given Romanelli and colleagues’ (2022) findings that Black and male adolescents are especially likely to report attempted suicide without thoughts or plans, making it more difficult for traditional screening systems to identify these high-risk youth. Other patterns, such as the presence or absence of non-suicidal self-injury, could also be relevant (Stewart et al., 2017).

The need for research on group differences, novel risk factors of suicide, and prediction methods has been repeatedly highlighted in recent reviews (Cha et al., 2018; May & Klonsky, 2016; Nock et al., 2016). The current study provides one investigation of demographic, home, school, and mental health factors differentiating youth with varying levels of suicidal thoughts and behavior. Continued research establishing robust links between these patterns of suicidal thoughts and behaviors and demographic, home, school, and mental health factors could be used to inform triage. However, in clinical practice, suicide assessment usually relies on single-domain suicide measures (e.g., the Columbia-Suicide Severity Rating Scale [CSSRS], the ASK Suicide-Screening Questions; Horowitz et al., 2012; Posner et al., 2011), thus limiting the potential for differentiating these subgroups based on risk factors. Using broadband screeners like the BHS, which inquire about these biopsychological risk factors that are usually only collected in a larger research study, could lead to more effective clinical decision-making. Given advancements in analytic modeling (e.g., latent class analysis, the p-factor, machine learning; Burke et al., 2020; Diamond et al., 2017; Herres et al., 2018; Ruan-Iu et al., 2022a, 2022b), risk algorithms could be incorporated into a web-based reporting system, such as bhworks, to inform clinical decision-making regarding triage. This line of research has the potential to improve behavioral health systems and slow the accelerating rates of adolescent suicide.

## Data Availability

The data and materials used for this study are not available.

## Code Availability

The code is available upon request.
